# The dual roles of human PYHIN family proteins in cancer: mechanisms and therapeutic implications

**DOI:** 10.3389/fimmu.2025.1576674

**Published:** 2025-05-02

**Authors:** Shuyan Zeng, Zhiyong Zhou, Yi Li, Di Wu, Qiuyun Xiao, Huiyun Peng

**Affiliations:** ^1^ Center for Molecular Diagnosis and Precision Medicine, The First Affiliated Hospital of Jiangxi Medical College, Nanchang University, Nanchang, China; ^2^ Huankui Academy, Nanchang University, Nanchang, China; ^3^ Jiangxi Provincial Center for Advanced Diagnostic Technology and Precision Medicine, The First Affiliated Hospital of Jiangxi Medical College, Nanchang University, Nanchang, China

**Keywords:** human PYHIN family, AIM2, IFI16, IFIX, MNDA, inflammasome, tumor suppressor, tumor promoter

## Abstract

The human PYHIN family proteins, including AIM2, IFI16, IFIX, and MNDA, which are crucial cytosolic nucleic acid sensors. These proteins share a common structural feature, including signature N-terminal PYD domain and C-terminal HIN-200 domain, which enable them to recognize intracellular nucleic acids and assemble inflammasomes, triggering inflammatory responses and programmed cell death. Over the last decade, it has emerged that the PYHIN family proteins play multifaceted roles in cancer biology, with dualistic roles due to tumor heterogeneity and the tumor microenvironment’s plasticity through dependent or independent of inflammasome mechanisms. Here, we discuss their ability to function as both a tumor suppressor and a tumor promoter of tumor progression emphasizes the need for further research to delineate the precise mechanisms by which these proteins operate in various cancer contexts. Understanding these dynamics could pave the way for novel therapeutic approaches that harness the dual nature of PYHIN family members to improve cancer treatment outcomes.

## 1 Introduction

The innate immune system is the first defense line that recognizes pathogen-associated molecular patterns (PAMPs) and damage-associated molecular patterns (DAMP) by pattern recognition receptors (PRRs) against pathogen incursion. Membrane-bound PRRS include Toll-like receptors (TLR) and C-type lectin receptors (CLR), while cytoplasmic PRRS include NOD-like receptors (NLR), RIG-1 like receptors (retinoic acid-induced gene-I receptor, RLRs), and PYHIN (pyrin and HIN domain-containing proteins) family members (1). The specific ligands of these receptors and the mechanisms of their signaling cascades have been relatively well explored. With the deepening of the research on immune response caused by DNA in cells, four members of human PYHIN family have been discovered, including absent in melanoma 2 (AIM2), IFN-inducible protein 16 (IFI16), IFN-inducible protein X (IFIX/PYHIN1), myeloid cell nuclear differentiation antigen (MNDA) (2), which have similar molecular structures featuring an N-terminal pyrin domain (PYD) and a C-terminal oligonucleotide-binding HIN-200 domain (hematopoietic, interferon-inducible nuclear protein with 200 amino acid repeat) (3). The PYD domain facilitates protein-protein interactions, particularly with the ASC (apoptosis-associated speck-like protein containing a caspase recruitment team), which is essential for inflammasome formation and activation. The HIN-200 domain, on the other hand, is responsible for binding to DNA, enabling these proteins to sense and respond to the presence of foreign or damaged nucleic acids. Functionally, human PYHIN family can be categorized into DNA sensors that mediate the production of type I IFN and those that mediate inflammasome activation. Despite their shared family lineage, these proteins exhibit distinct structural and functional differences that influence their roles in immunity.

Members of the human PYHIN family not only participate in the formation and activation of inflammasomes but may also function through non-inflammasome-dependent mechanisms influencing inflammation and tumor progression in different contexts. AIM2 and IFI16, for instance, are well-known for their roles in forming the inflammasome, which is crucial for the activation of caspase-1 and the subsequent release of pro-inflammatory cytokines like IL-1β. The activation of AIM2 is closely associated with the onset and progression of various diseases, particularly in inflammatory bowel disease (IBD), where AIM2 expression is significantly upregulated, suggesting its potential role in disease progression (4). Studies have shown that AIM2 can promote tumor growth in KRAS-driven lung adenocarcinoma models, indicating its role in tumorigenesis independent of inflammasome activation. This process is particularly relevant in the context of cancers where inflammation is a driving factor. Several non-inflammasome-independent pathways involved in neoplastic development, including cellular senescence (5), regulation of gene expression (6, 7), modulation of immune signaling (8), DNA damage responses (9), and cell cycle regulation (10). Additionally, IFI16 has been shown to suppress the activation of other inflammasomes, such as AIM2 and NLRP3, thereby modulating inflammatory responses in a way that can either promote or inhibit tumor progression depending on the context (11). On the other hand, MNDA, another member of the PYHIN family, has been implicated in the regulation of gene transcription and immune responses. Its ability to bind DNA and regulate transcription in monocytes suggests that MNDA may also play a role in modulating inflammation and tumorigenesis through non-inflammasome pathways (12). The structural insights into MNDA’s interaction with DNA reveal a unique binding mode that could influence its function in immune regulation and cancer biology.

The interplay between these PYHIN proteins and their mechanisms of action underscores the complexity of their roles in inflammation and tumor progression. While inflammasome activation is a well-characterized pathway, the non-inflammasome functions of these proteins are gaining recognition for their potential impact on tumor biology. Understanding these dual roles could provide new therapeutic avenues for targeting inflammation in cancer treatment, particularly in tumors characterized by aberrant immune responses. In this review, we commence with activation and immune regulation mechanisms of the human PYHIN family. Subsequent to this, our discussion centers on the diverse roles of AIM2, IFI16, IFIX, and MNDA, providing a comprehensive elucidation regarding their functions and mechanisms across various tumor types. Human HIN-200 proteins exhibit multifaceted roles in tumor biology in a context-dependent manner. Their intricate interplay in immunity and cancer progression paves the path for the potential as therapeutic targets.

## 2 Overview of human PYHIN family

### 2.1 Structure of human PYHIN family

**Figure 1:**
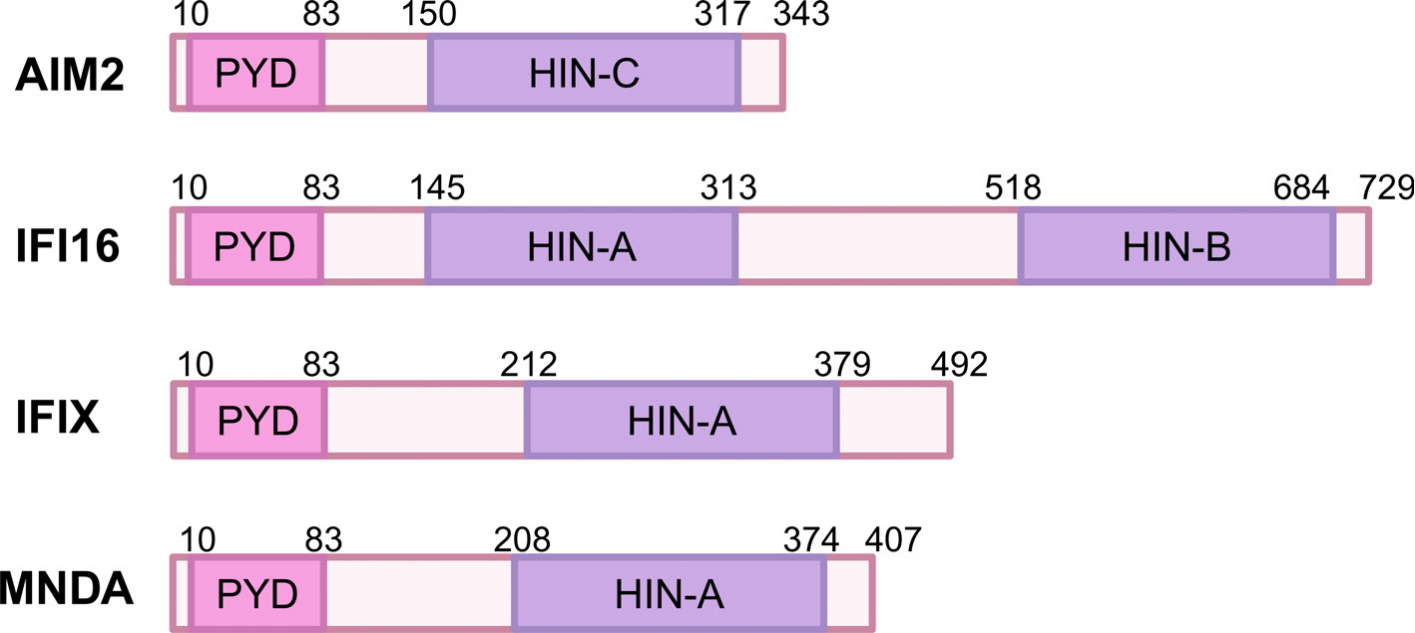
Domain organization of human PYHIN family proteins. The PYHIN protein has a conserved domain structure consisting of an N-terminal PYD and one or more C-terminal DNA binding HIN domains. PYD and HIN-200 are depicted in orange and red, respectively. The HIN domain is divided into three subtypes: HIN-A, HIN-B, and HIN-C. NCBI reference sequences are as follows: NP_004824.1 (AIM2); NP_001193496.1 (IFI16); NP_689714.2 (IFIX); NP_002423.1 (MNDA).

The human PYHIN family proteins, including AIM2, IFI16, IFIX, and MNDA, share a common structural framework characterized by an N-terminal PYD domain and a C-terminal HIN-200 domain (**Figure 1**). These domains play critical roles in the proteins’ ability to recognize intracellular nucleic acids and initiate immune responses. The PYD domain, also called Pyrin, PAAD or DAPIN, can interact with other PYD-containing family members or adapter protein ASC (13). The HIN domain consists of two consecutive oligonucleotide/oligosaccharide-binding (OB) folds and is classified into three subtypes: A, B, C (14). MNDA and IFIX contain a single type A HIN domain (HIN-A), IFI16 have one HIN-A and one HIN-B domain, while AIM2 possesses a single HIN-C domain. Through electrostatic attraction, the positively charged HIN domain senses and binds to the phosphoric groups of negatively charged single-stranded DNA (ssDNA) and double-stranded DNA (dsDNA) (14, 15).

First, from the perspective of the PYD domains, both AIM2 and IFI16’s PYD domains are involved in interacting with ASC to form inflammasome which activates caspase-1 and induces cell death (16, 17). The PYD domain of IFI16 promotes DNA binding and aggregation after binding to DNA, forming signaling aggregates that enhance antiviral response (18). In contrast, the PYD domain of IFIX plays a crucial role in regulating cell proliferation and apoptosis, but its specific role in the formation of inflammasome is not yet clear (19). In terms of HIN domains, both AIM2 and IFI16’s HIN domains can recognize dsDNA and bind to the sugar-phosphate backbone of DNA through electrostatic attraction (11, 20, 21). However, after binding to dsDNA, AIM2’s HIN domain releases its self-repressive state, thereby promoting the assembly of inflammasomes (22). In contrast, IFI16’s HIN domain forms a polymer upon recognizing DNA, enhancing its ability to sense viruses (23). The MNDA’s HIN domain also exhibits the capacity to engage with dsDNA through two distinct binding interfaces: AIM2 HIN-like DNA binding mode and a MNDA-specific DNA binding mode (12, 24), with the MNDA-specific mode differing from other HIN-200 proteins by being located opposite the AIM2-like binding interface, but its function within cells is primarily associated with regulating immune responses, and its role in inflammasomes remains unclear (24).

The differences in the PYD and HIN200 domains among AIM2, IFI16, IFIX, and MNDA not only reflect their evolutionary adaptations but also underscore their specialized functions in the immune system. Understanding these distinctions is crucial for developing targeted therapies that can modulate immune responses in various diseases, including infections, autoimmune disorders and cancers. The interplay between these proteins and their structural features continues to be an important area of research in immunology.

### 2.2 Distribution and function of human PYHIN family

**Figure 2:**
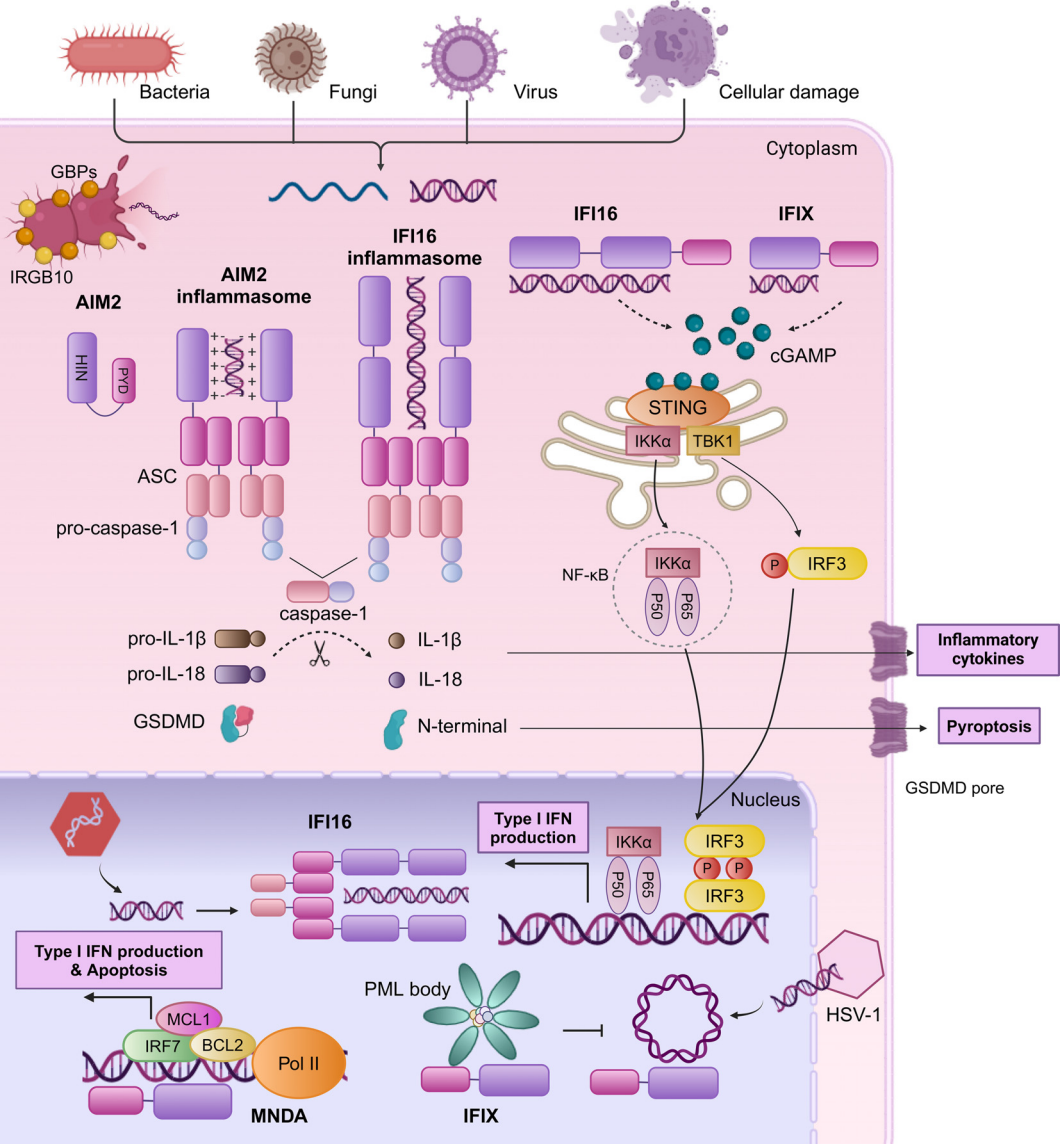
Human PYHIN family proteins sense dsDNA by mediating inflammasome complex and type I IFN signaling. AIM2 possesses a HIN domain that binds dsDNA and a PYD that interacts with the PYD of ASC. The CARD domain of ASC can then interact with the CARD domain of pro-caspase-1 to facilitate the maturation of IL-1β, IL-18, and GSDMD, culminating in the secretion of inflammatory factors and induction of pyroptosis. Upon DNA encounter within the nucleus, IFI16 initiates the formation of an ASC-dependent inflammasome complex, facilitating its translocation to the cytoplasm. In the cytoplasm, the HIN domain of IFI16 binds dsDNA to activate the cGAS-STING pathway through the elevation of cGAMP levels. This activation leads to STING-mediated IRF3 phosphorylation and NF-κB complex assembly, prompting their nuclear translocation to stimulate the expression of IFN-b and other immune-regulatory molecules. IFIX engages in a STING-TBK1-IRF3 response similar to IFI16 in the cytoplasm. During HSV-1 infection, IFIX recognizes and binds viral DNA within the nucleus, potentially through its recruitment to PML bodies. MNDA undergoes cleavage by caspase-1 during genotoxic reactions, leading to the translocation of the PYD fragment to the cytoplasm, where is hypothesized to promote inflammasome assembly (not illustrated). MNDA interacts with chromatin and RNA PolII to control the transcription of genes such as IRF7, MCL1, and BCL2, enhancing production of type I IFN and apoptosis.

Nucleic acid sensing by PYHIN family proteins includes dsDNA, ssDNA, and DNA-RNA hybrids, primarily derived from microbial pathogens and endogenous cancer or necrotic cells. During bacterial infections, IFN-inducible proteins like guanylate-binding proteins (GBPs) and immunity-related GTPase family member b10 (IRGB10) facilitate the release of bacterial DNA into the cytoplasm (25, 26). In cancer cells, DNA originating from cell apoptosis, active secretion, and exosome release is particularly prominent in cytoplasm due to genomic instability, necrosis. Furthermore, ionizing radiation and chemotherapeutic agents can induce double-strand DNA breaks, with this DNA primarily located in the nucleus. In the study of intracellular DNA sensors, proteins such as AIM2, IFI16, IFI204, and MNDA are particularly important for their distribution in the cytoplasm and nucleus and their ability to recognize different DNAs (**Figure 2**). Except for AIM2, primarily cytoplasmic, other members contain at least one multipartite N-terminal nuclear localization signal (NLS) and can translocate from the nucleus to the cytoplasm under pathogen DNA stimulation (6, 27–29).

Human PYHIN family proteins initiate immune responses through two pathways: inflammasome assembly/activation and type I IFN production. One pathway is the responses of AIM2 and IFI16 to Kaposi sarcoma-associated herpesvirus (KSHV) and herpes simplex virus 1 (HSV-1) infections (30–32), which bind to the sugar backbone of cytosolic dsDNA through its positively charged HIN domain and then associates with ASC via PYD-PYD interactions, recruit pro-caspase-1 through caspase recruitment domain (CARD)-CARD interactions that converted into active caspase-1, and then it catalyzes the cleavage of pro-IL-1β, pro-IL-18, and Gasdermin D (GSDMD), leading to the maturation and secretion of inflammatory factors IL-1β, IL-18, and the formation of GSDMD pores on the cell membrane, inducing pyroptosis. The production of inflammatory factors and pyroptosis trigger a series of innate immune mechanisms to clear pathogens. However, the progress of immune responses can be deactivated by certain viral tegument proteins, such as pUL83 of human cytomegalovirus (HCMV) and VP22 of HSV-1 (33, 34). Furthermore, elevated levels of POP3 or IFI16 also suppress the activation of AIM2 inflammasomes, possibly due to competitive recruitment of ASC (35). Concurrently, the other pathway is responsible for IFI16 and IFIX distinctive as a dual sensor for both nuclear and cytoplasmic dsDNA to mediate type I IFN production through the stimulator of interferon genes (STING) -TANK binding kinase 1 (TBK1) -IFN regulatory factor 3 (IRF3) signaling cascade. Ultimately, type I IFN plays a central role in cross-priming, T cell activation, and tumor regression. In HSV-1-infected cells, IFI16 binds to the viral DNA at the nuclear periphery (36). Subsequently, phosphorylation of the intrinsically disordered region leads to IFI16 oligomerization and the extension into a solid filamentous structure (36). Then, IFI16 translocates to the cytoplasm, resulting in the recruitment of IFI16-STING-mediated TBK1, the activation of the IRF3 and NF-κB pathways, and the generation of IFN-β (28). IFIX engages in a STING-TBK1-IRF3 response similar to IFI16 in the cytoplasm, playing a crucial role in DNA damage response pathways.

Beyond the inflammasome complex and type I IFN signaling, the human PYHIN family proteins have been found to engage in a variety of mechanisms. IFIX binds HSV-1 DNA substrates in a sequence-independent manner, potentially through its recruitment to subnuclear protein structures known as PML (promyelocytic leukemia) bodies (37). MNDA interacts with chromatin and RNA polymerase II (Pol II) to control the transcription of genes such as IRF7, myeloid cell leukemia 1 (MCL1), and BCL2, enhancing type I IFN production (38) and promoting apoptosis in leukocytes and chronic lymphocytic leukemia (CLL) cells (6, 7). Additionally, IFI16 inhibits genes transcription by interfering with transcription factors (39), impeding their binding cognate promoters (40) or modulating chromatin structure (40–42). Specifically, IFI16 disrupts the binding of TATA-binding protein (TBP) and octamer-binding transcription factor 1 (Oct 1) to the HSV-1 promoter (40), and directly interacts with Sp1 to suppress HIV-1 transcription (39). Additionally, IFI16 induces the chromatinization of viral DNA, thereby suppressing the replication and expression of viral genes (43).

In summary, the distribution and functional differences of these DNA sensors enable them to play their respective roles under different physiological and pathological conditions. This complex regulatory mechanism allows cells to effectively respond to exogenous DNA threats and also provides new insights into host immune responses. These studies provide a theoretical foundation for the future development of therapeutic strategies targeting viral infections, autoimmune diseases and cancers.

## 3 The dual role of AIM2 in cancer

### 3.1 The anti-tumor role of AIM2

#### 3.1.1 AIM2 in glioblastoma multiforme

AIM2 is one of the tumor-associated antigens expressed by glioma cells, which specifically recognize and engage with the HLA-A1 locus (44). Due to this property, AIM2 is commonly applied to the monitoring test of new dendritic cell vaccine and immunotherapy which can attack and destroy tumor cells (45, 46). There was a significant inverse correlation between methylation levels of CpG loci and AIM2 gene expression in GBM, with distinct variations observed based on the tumor grade (47). Notably, AIM2 expression was an increase in spatial heterogeneity within GBM samples, with strong expression in tumor cores associated with reduced cell proliferation (**Table 1**) (48). In astrocytoma, the expression of AIM2 induced by the inflammatory agent Neamycin was accompanied by decreased cell proliferation and mitochondrial activity. In general, these observations indicated that AIM2 was independent of inflammasome function (48), suggesting that AIM2 might be a reasonable regulator of GBM cell proliferation.

**Table 1:** The role of AIM2 in tumor biology and the underlying mechanisms in various tumor types.

Tumor types	Molecular mechanisms of AIM2	System	Role of AIM2	References
GBM	Inhibition of cell proliferation independent of the inflammasome with IL-1β secretion	Brain specimens from patients with epilepsy and U251 cells	Anti-tumor	(48)
SCC	Activation of NF-κB signaling	OSCC tumors samples; human OSCC cell lines (Ca9-22, HO-1-u-1, HSC-2, HSC-3, HSC-4, SAS, HSQ-89, Sa-3)	Pro-tumor	(93)
	Promoting NF-κB/BCL2A1/MAPK/c-Myc pathway	PSCC tumors samples; Penl2, 149rca and 156lm PSCC cell lines	Pro-tumor	(96)
	Activation of STAT1/NF-κB and promotion of irradiation resistance, migration ability and PD-L1 expression	Oral cancer cell lines HSC2, HSC3, HSC4 and SAS	Pro-tumor	(95, 177)
	STAT1 mediated transcription and IL-17/MAPK signaling	HNSCC specimens and HNSCC cell lines (2A3 LCC, CAL27, FADU and HEK 293 T, BEAS-2B)	Pro-tumor	(91)
NSCLC	Facilitation of calcium release and mitochondrial ROS generation, resulting in calpain activation and elevated IL-1α concentrations	Lung samples of NSCLC patients; pDCs	Pro-tumor	(111)
	Fostering M2 polarization and PD-L1 expression through the JAK/STAT3 pathway	Lung samples of NSCLC patients, C57BL/6N mice	Pro-tumor	(108, 109)
	Regulation of cell-cycle, induction of G2/M phase arrest	BALB/c athymic nude mice, Human NSCLC A549, H460, H226 and 16HBE	Pro-tumor	(102, 178)
	Augmenting ROS generation and activating the MAPK/ERK signaling	Human H1975, H358, A549, H157, HCC827, H3255, and H460 NSCLC cell lines, Xenograft tumors	Pro-tumor	(103)
	Promoting PD-L1 expression and immune evasion of NSCLC via NF-κB/STAT1 and JAK/STAT3 pathway	Lung adenocarcinoma cell lines A549, H1355, HCC827, and PC9; Raw264.7, LA795 cells and C57BL/6J-Aim2em1Cya mice	Pro-tumor	(101, 109)
	Inhibition of ferroptosis through STAT5B-ACSL4 axis	Lung cancer cell lines NCI-H1395, SK-MES-1, and NCI-H1975	Pro-tumor	(179)
BC	Inhibition of NF-κB transcriptional function and reduced sensitivity to TNFα-driven NF-κB activation	MCF-7, MDA-MB-231, MDA-MB-435, and MDA-MB-453 cells; tetracycline-inducible AIM2 Cell Lines	Anti-tumor	(51)
	Down-regulating Bcl-xl, up-regulating Bad and Bax, activating caspases that leads to the cleavage of PARP	MCF-7 tTA-AIM2 cells, MCF-7 tTA-Luc cells under Tet-Off model system	Anti-tumor	(52)
	Promoting the division of DFNA5 by activating caspase-3	BALB/c nude mice and MCF-7, MDA-MB-231 cell lines	Anti-tumor	(53)
HCC	Inhibiting the mTOR-S6K1 signaling pathway and in turn inhibiting HIF-1α	BALB/c athymic nude mice and HCC cell lines (MHCC97H, MHCC97L, BEL7402, SMCC7721, HepG2 and HUH7)	Anti-tumor	(54)
	Promote autophagy and inhibiting M2 macrophage polarization	Normal hepatocyte(L-02) and HCC cells (Huh-7, Hep3B, and PLC/PRF/5); HepG2215 cells	Anti-tumor	(55, 59)
	Binding and reduction influenced by HBx; reduction of AIM2 stimulates EMT by elevating FN1 expression	Primary HCC patients and HCC cell lines (Bel-7402, SMMC-7721, Huh7 and Bel-7404)	Anti-tumor	(57)
GC	Inhibition the proliferation and migration of GC cells through the inhibition of Akt signaling pathway	GC specimens; SGC7901 GC cells	Anti-tumor	(63)
	Interaction with EB1 and connection of the cytokine-STAT3 signaling pathway	GC cell line xenografts and gp130 F/F mice	Pro-tumor	(118)
	Increase of the phosphorylation of p38, JNK and ERK and promotion of the MAPK signaling to promote proliferation and migration	BGC-823 and MGC-803 GC cells	Pro-tumor	(117)
RCC	Inhibition of cell invasion and metastasis via enhancing autophagy induction.	786-O and OSRC-2 RCC cells	Anti-tumor	(68)
	Promoting M1 polarization to inhibit malignant behaviors of tumor cells	BALB/c mice and RCC cell lines (HEK293T, L929)	Anti-tumor	(180)
	Inhibition of ferroptosis through FOXO3a-ACSL4 axis	RCC cell lines (786O, OSRC2, Caki-1, A498 and ACHN) and renal tubular epithelial cell line HK-2	Pro-tumor	(116)
CRC	Initiating G2/M cell cycle halt, protracted progression from G2 to M phase in the cellular cycle	HCT116 CRC cells	Anti-tumor	(10, 72, 76)
	Inhibition of cell growth and modulation of intestinal stem cell proliferation	Wild-type and AIM2-deficient mice, organoid models, and bone marrow chimera constructs	Anti-tumor	(75)
	Interaction with DNA-PK and Inhibition of Akt activation	Wild-type and AIM2-deleted mice, MEFs, BMDM models, and organoid systems	Anti-tumor	(74)
	Promoting apoptosis in CRC cells by suppressing PI3K/Akt pathway	HCT116 CRC cells	Anti-tumor	(72)
	Regulating Gli1 to inhibit proliferation and migration through the Akt/mTOR pathway	HCT116、SW480、SW620 and LoVo CRC cells	Anti-tumor	(73)
	Inhibition of BRAF-mutant CRC growth in a caspase-1-dependent manner	50 CRC patients and CRC cell lines (HCT29, HCT116, COLO205, and SW480)	Anti-tumor	(79)
	Inhibition of proliferation and migration via P38MAPK signaling	HCT116 CRC cells	Anti-tumor	(10)
PCa	Gathering of senescent cells in the prostate epithelium	Human prostate cancer cell lines (RWPE-1, RWPE-2, DU-145, PC-3, and LNCaP)	Anti-tumor	(86)
OS	Inhibition of PI3K/Akt/mTOR signaling pathway and EMT	OS cell lines C396, CAL-72 and MG-63	Anti-tumor	(88)

#### 3.1.2 AIM2 in EBV-associated nasopharyngeal carcinoma

Both AIM2 and IL-1β exhibit elevated expression in EB virus-associated NPC, with significant correlations between their expression levels and patients’ survival (49). The activation of the AIM2 inflammasome by viral stimuli and elements within tumor microenvironment (TME) facilitates the secretion of IL-1β. Intriguingly, tumor-derived IL-1β has been implicated in enhancing local tumor control and improved patient survival (49), indicating a paradoxical role for the tumor inflammasome in tumor suppression. The precise interplay between AIM2 and IL-1β in NPC, particularly how they synergize to inhibit tumor progression, warrants further investigation.

#### 3.1.3 AIM2 in squamous cell carcinoma

The prognostic significance of AIM2 in SCC is underscored by studies showing that low AIM2 expression is associated with poor survival outcomes. In hypopharyngeal squamous cell carcinoma, for instance, the expression of AIM2 in tumor tissues was significantly lower than that in adjacent normal hypopharyngeal tissues. Low AIM2 levels combined with high expression of p-STAT3, correlate with increased lymph node metastasis and poor prognosis, highlighting its potential as a biomarker for disease progression (**Table 1**) (50). Therefore, AIM2 may represent a valuable prognostic marker and therapeutic target in SCC, warranting further investigation into its role in tumor progression and metastasis.

#### 3.1.4 AIM2 in breast cancer

**Figure 3:**
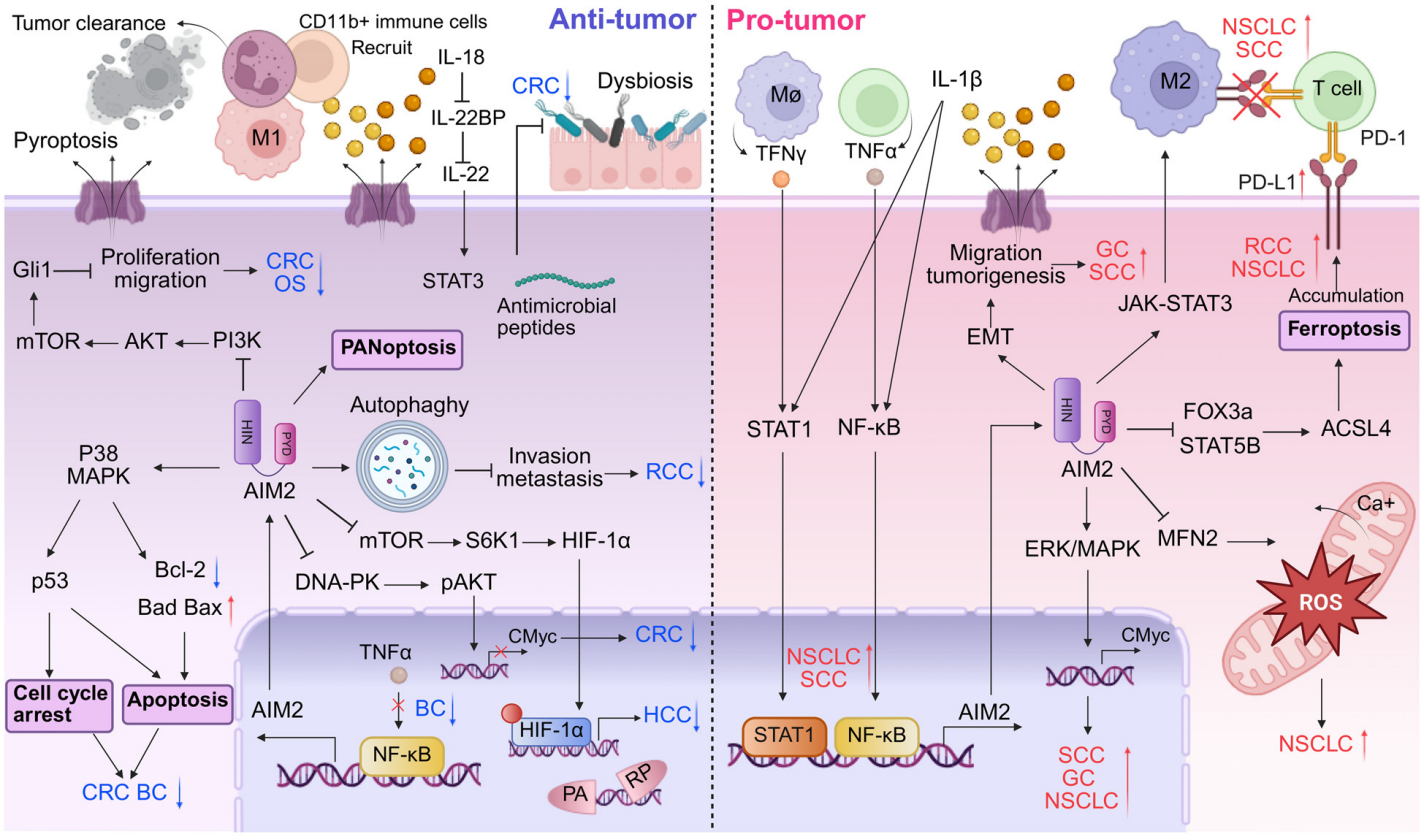
Dual roles of AIM2 in tumor microenvironment. AIM2 exhibits a bivalent role in tumor progression by modulating both pro- and anti-oncogenic activities within the immune landscape. In TME where AIM2 exerts tumor-suppressive functions, it facilitates the recruitment of inflammatory immune cells, such as T cells and NK cells, to promote tumor clearance. Furthermore, AIM2 enhances cellular death mechanisms including apoptosis, autophagy, and PANoptosis. AIM2 can also inhibit tumor growth via various inflammasome-independent pathways, notably in CRC. Conversely, in tumor contexts where AIM2 exerts oncogenic effects, it fosters an immunosuppressive environment marked by M2 macrophage polarization and upregulation of PD-L1, thereby dampening the immune response against tumors and promoting immune evasion. Additionally, AIM2 inflammasome-mediated release of pro-inflammatory cytokines like IL-1β can activate signaling cascades that enhance tumor growth and metastasis. While cell death mechanisms such as ferroptosis are attenuated, processes that facilitate cell migration, including EMT, are augmented.

Research has illuminated that the expression of AIM2 can suppress the proliferation and tumorigenicity of human breast cancer cells through the negative regulation of the TNF-α/NF-κB anti-apoptotic pathway (**Figure 3**, left panels) (51). Liu et al. revealed that the expression of AIM2 leads to the cytoplasmic translocation of cytochrome C, simultaneously with downregulation of the Bcl-xl, upregulation of Bax, culminating in the cleavage of PARP (**Figure 3**, left panels) (52). In addition, AIM2 enhances the cleavage of DFNA5 by activating caspase-3, and that docosahexaenoic acid (DHA) triggers pyroptosis in BC cells through the pivotal AIM2/Caspase-3/DFNA5 pathway (53). In summary, these studies collectively shed light on AIM2’s multifaceted role in the regulation of BC cells death, presenting new therapeutic opportunities in oncology.

#### 3.1.5 AIM2 in hepatocellular carcinoma

In an intriguing analysis of the molecular landscape of liver cancer, the expression profile of AIM2 has emerged as a pivotal determinant of tumor progression. Notably, a significant downregulation of AIM2 expression was observed in HCC tissues (**Figure 3**, left panels and **Table 1**) (54), with this decremental trend closely associated with advanced stages of tumorigenesis. Further investigation revealed that exogenous overexpression of AIM2 in HCC cells exerted inhibitory effects on the mammalian target of rapamycin (mTOR)-S6K1 signaling pathway, a critical axis implicated in cellular proliferation, survival, and metabolism (54). Consequently, this upregulation of AIM2 led to a suppression of HCC cells proliferation, colony formation, and invasive capabilities, highlighting its tumor-suppressive role. The expression of caspase-1 and the levels of IL-1β and IL-18 are positively correlated with AIM2 expression in HCC cells. Activation of the AIM2 inflammasome has been shown to promote autophagy and inhibit M2 macrophage polarization, potentially through the secretion of inflammatory cytokines (55). AIM2’s role in amplifying autophagy also extends to mitigating age-related acute liver injury (56). Specifically, AIM2 has been implicated in regulating lipid peroxidation and oxidative stress in aged mice, highlighting its protective role in acute liver damage (56). Of particular note, the loss of AIM2 induced by Hepatitis B virus X protein (HBx) has been implicated in adverse clinical outcomes and facilitated HCC metastasis through the induction of EMT (57), a process pivotal for cancer cell dissemination and colonization at distant sites.

In the therapeutic realm, radiofrequency ablation (RFA) has demonstrated efficacy inhibit HCC tumor growth by harnessing the AIM2-mediated induction of pyroptosis, a lytic form of programmed cell death (58). This mechanism suggests that therapeutically targeting AIM2-driven inflammasome signaling pathways may offer a promising strategy to augment the therapeutic outcomes of RFA in HCC patients. Moreover, a novel mechanism has been unveiled wherein dihydroartemisinin, a compound derived from traditional medicinal plants, promotes the activation of the AIM2/caspase-1 inflammasome, thereby contributing to autophagy in HepG2215 cells (59). This discovery broadens the therapeutic horizon for HCC by suggesting potential synergistic effects between dihydroartemisinin and AIM2-mediated pathways in fostering cellular self-digestion, a process known to counteract tumorigenesis. In advanced HCC, a recent study has revealed that in HCC cells overexpressing DANSE1L3, sorafenib induces pyroptosis, apoptosis, and necroptosis (PANoptosis) rather than apoptosis (60). Unlike apoptosis, PANoptosis is mediated by AIM2 and leads to the release of pro-inflammatory cytokines, which enhances immunogenicity and transforms “immune-cold” tumors into “immune-hot” tumors, thereby increasing the efficacy of immunotherapy (61). This finding underscores the potential of targeting AIM2 to modulate PANoptosis as a novel strategy for enhancing immunotherapy in HCC.

Collectively, these findings underscore the multifaceted roles of AIM2 in HCC biology and its potential as a therapeutic target. Future studies exploring the intricate interplay between AIM2 and its downstream effectors, as well as the integration of AIM2-targeting strategies into existing treatment paradigms, hold promise for advancing the management of HCC.

#### 3.1.6 AIM2 in gastric cancer

Reports on the role of AIM2 in GC have yielded mixed results and we will delve into its tumor-suppressing effects. Zhou et al. compared the samples of patients with early and advanced GC and demonstrated that AIM2 expression was notably lower in advanced GC tissues (62). Mechanistically, AIM2 exerts its anti-tumor effects by regulating the Akt signaling pathway. The knockdown of AIM2 in GC cells leads to increased cellular proliferation and migration, whereas its overexpression results in the opposite effects, highlighting its potential as a tumor suppressor in GC (**Table 1**) (63). Targeting the AIM2/Akt signaling axis could offer new avenues for GC treatment, providing a novel entry point for therapeutic intervention.

#### 3.1.7 AIM2 in renal cell carcinoma

In the context of RCC, AIM2 has been recognized as a potential biomarker and therapeutic target. Chai et al. practiced combined molecularly targeted anti-cancer therapies and immunotherapies, developing nanoparticle systems to deliver AIM2 (64–66). Furthermore, the role of AIM2 in RCC is linked to its interaction with other molecular pathways. For instance, the Wnt/β-catenin signaling pathway (67) and upregulating autophagy-related genes (Bcl-2, Beclin-1 and ATG-5) (**Table 1**) (68), have been implicated in RCC progression, and AIM2’s influence on these pathways could provide insights into its anti-tumor role (**Figure 3**, left panels). The modulation of these pathways by AIM2 could potentially inhibit RCC cell proliferation, invasion, and migration, offering a novel therapeutic angle. In addition to its direct effects on tumor cells, AIM2’s role in regulating immune responses is of particular interest. The cytokine interleukin-18 (IL-18), which has immunostimulatory effects, is negatively regulated by IL-18 binding protein (IL-18BP). A decoy-resistant form of IL-18 (DR-18) has been developed to enhance its anti-cancer efficacy. In RCC, DR-18 has been shown to enhance the activity of anti-CTLA-4 treatment, suggesting that AIM2’s modulation of immune pathways could be leveraged to improve immunotherapy outcomes (69).

Overall, AIM2’s involvement in RCC highlights the complexity of cancer biology and the need for integrated approaches to treatment. By targeting AIM2 and its associated pathways, there is potential to develop more effective therapies for RCC, improving patient outcomes and overcoming resistance to current treatments.

#### 3.1.8 AIM2 in colorectal cancer

The AIM2 protein has emerged as a significant player in the context of CRC, particularly due to its role as a DNA sensor in the innate immune response. Recent studies have highlighted AIM2’s potential as a tumor suppressor, demonstrating its involvement in regulating various cellular processes that are crucial for tumor progression and metastasis. For instance, AIM2 has been shown to inhibit epithelial-mesenchymal transition (EMT) in CRC cells, a process that is often associated with increased invasiveness and metastatic potential. This suppression of EMT is mediated through the Akt signaling pathway and the inflammasome pathway, indicating that AIM2 may exert its anti-tumor effects by modulating critical signaling cascades involved in cancer cell behavior (**Figure 3**, left panels) (70–73). Several research revealed that AIM2 inhibits the proliferation of CRC cells by interacting with DNA-dependent protein kinase DNA-PK (74, 75) or triggering cell cycle arrest (**Figure 3**, left panels) (10, 72, 76). Furthermore, AIM2’s expression levels have been correlated with patient prognosis in CRC. Low levels of AIM2 have been associated with poorer outcomes, suggesting that it may serve as a valuable prognostic marker (77, 78). In BRAF-mutant CRC, restoring AIM2 expression significantly inhibited tumor growth and induced necrotic cell death in a caspase-1 dependent manner, further underscoring its role as a tumor suppressor (**Figure 3**, left panels) (79). The interplay between AIM2 and the tumor microenvironment also warrants attention, as AIM2 has been implicated in enhancing immune responses against tumor cells, potentially through the activation of pro-inflammatory cytokines like IL-1β (80).

In addition to its direct effects on tumor cells, AIM2’s interaction with the gut microbiota has been identified as a critical factor in CRC susceptibility. Dysbiosis in the gut microbiota can exacerbate the tumorigenic effects of AIM2 deficiency, highlighting the complex relationship between host genetics, immune responses, and microbial influences in CRC development (74, 75). Moreover, the AIM2 inflammasome plays a central role in preventing gut microbiota dysbiosis and intestinal inflammation by modulating the STAT3 pathways and the expression of specific antimicrobial peptides (**Figure 3**, left panels) (81). This synergy suggests that therapeutic strategies aimed at modulating AIM2 expression or enhancing its activity could provide new avenues for CRC prevention and treatment. Moreover, the therapeutic potential of targeting AIM2 in CRC is supported by its ability to regulate the expression of key immune-related genes. AIM2 has been shown to mediate the expression of HLA-DRA and HLA-DRB, which are crucial for antigen presentation and immune surveillance (71). This indicates that AIM2 not only plays a role in tumor suppression but also in shaping the immune landscape of CRC, making it a promising target for immunotherapy approaches (**Table 1**).

Overall, AIM2 represents a multifaceted target in CRC, with its anti-tumor effects stemming from its ability to regulate cell signaling, influence the immune microenvironment, and interact with gut microbiota. Continued research into the mechanisms underlying AIM2’s functions could pave the way for novel therapeutic strategies aimed at improving outcomes for CRC patients.

#### 3.1.9 AIM2 in HPV-infected cervical cancer

HPV is a sexually transmitted DNA virus that has been irrevocably linked to the development of CCA (82). During the early stages of tumorigenesis, AIM2 expression might ascend in response to viral infection and declined at a later stage due to various inhibitory mechanisms. SIRT1 is frequently overexpressed in HPV-infected CCA, with a negative regulatory effect on the transcription of the AIM2 gene. This regulation impaired the AIM2 inflammasome and enable HPV-infected CCA cells to evade antiviral immune responses (83). Furthermore, the knockout of SIRT1 has been shown to reactivate the AIM2 inflammasome, triggering the demise of CCA cells (83). Consequently, the interplay between SIRT1 and AIM2 may emerge as a promising therapeutic target for human CCA.

#### 3.1.10 AIM2 in prostate cancer

Extensive research correlates inflammation with the PCa development (84, 85). Expression of AIM2 mRNA is elevated in benign prostatic hyperplasia compared to normal prostate tissue, yet it is significantly diminished in PCa cells (86). The loss of AIM2 expression in PCa cells is associated with increased tumor growth and metastasis, highlighting its importance in maintaining cellular homeostasis and preventing malignant transformation. In addition, IFN treatment upregulate AIM2 in PCa cell lines, activating the AIM2 inflammasome and promoting IL-1β secretion (**Table 1**) (86). IL-1β is also known to support the progression of bone colonization and metastasis of forefront cancer cells (87). Currently, there is an absence of direct evidence linking AIM2’s inhibitory effects on PCa, and the underlying mechanisms remain elusive.

#### 3.1.11 AIM2 in osteosarcoma

AIM2 is down-regulated in OS cell lines, and its overexpression has been shown to exert a suppressive effect on the proliferation and migration, while simultaneously promoting apoptosis (**Table 1**) (88). Furthermore, it has been observed that the overexpression of AIM2 leads to a significant downregulation of the phosphorylation levels of key proteins within the PI3K/Akt/mTOR signaling pathway (**Figure 3**, left panels) (89, 90). These findings not only corroborate the tumor-suppressive role of AIM2 but also shed light on the molecular pathways that may be targeted for therapeutic intervention in OS.

### 3.2 The pro-tumor role of AIM2

#### 3.2.1 AIM2 in squamous cell carcinoma

Based on the bioinformatics analysis and immunohistochemistry, elevated levels of AIM2 are prevalent in primary oral squamous cell carcinoma (OSCC) tissues compared to adjacent normal tissues, which are positively associated with disease stage and HPV infection (91, 92). It has been noted that simultaneous high expression of AIM2 and IFI16 promoted p53-deficient OSCC cell proliferation by activating NF-κB signaling pathway, while they inhibited wild-type p53 cells growth (93). These results indicated that AIM2 possess oncogenic properties in OSCC cells with p53 deletion (93, 94). AIM2 overexpression has been shown to increase tumor growth and invasion into lymphatic vessels by promoting EMT that is a critical process for cancer metastasis, which lead to decreased survival rates in affected individuals (**Figure 3**, right panels) (92). AIM2 exerts pro-tumor effects activated by STAT1/NF-κB transcription through the IL-17/MAPK pathway (**Figure 3**, right panels) (91). In turn, upregulated AIM2 further activates STAT1/NF-κB signaling pathway through IL-1β and induction of immune cell infiltration that produces IFNγ and TNF-α, to promote irradiation resistance, migration ability and PD-L1 expression in OSCC (**Figure 3**, right panels) (95). Additionally, AIM2 are emerging as a valuable indicator for predicting the efficacy of immune checkpoint inhibitors against refractory OSCC (95).

In penile squamous cell carcinoma (PSCC), the upregulation of AIM2 and BCL2A1 is associated with pN status and can serve as a novel molecular classifier for precise prognostic assessment (96). Silencing AIM2 inhibits the growth of cancerous epithelial cells via an anti-tumor inflammatory cytokine-independent pathway (96). Moreover, knockdown of AIM2 significantly reduces BCL2A1 expression through the NF-κB pathway, thereby suppressing the MAPK/c-Myc signaling pathway (**Figure 3**, right panels and **Table 1**) (96).

The presence of AIM2 in cutaneous squamous cell carcinoma (cSCC) cells is more abundant than in normal skin tissues. Additionally, the reduction of AIM2 expression triggers cSCC cell apoptosis, diminishes cell invasiveness, and impedes cell vascularization (97). These findings indicate that AIM2 may function as a protumorigenic factor in cutaneous squamous cell carcinoma (cSCC), and that targeting AIM2 could potentially curtail tumor growth and invasive capacity. dihydroartemisinin, a compound with both antimalarial and antitumor capabilities (98), has been further discovered to exert effects by inhibiting the activation of AIM2 inflammasome pathway (99). These observations hint at the potential of AIM2 as a therapeutic target for SCC.

AIM2 has been identified as a potential biomarker for predicting tumor progression and patient outcomes in various SCC types. Its expression levels could serve as a prognostic factor, helping to stratify patients based on their risk of tumor progression and survival.

#### 3.2.2 AIM2 in non-small cell lung cancer

Cancer-elicited inflammation is a critical factor in the carcinogenesis and metastasis of NSCLC. AIM2 expression is frequently dysregulated in NSCLC, with increased levels observed in tumor tissues compared to normal lung tissue reducing overall survival (100, 101). Different studies have drawn different conclusions about the pro-cancer effects of AIM2 independent or independent of inflammasomes activation (102–104). Upon exposure to noxious substances, the AIM2 inflammasome is activated, thereby inducing sustained oxidative stress and lung injury (105). Additionally, AIM2 might influence the tumor microenvironment by affecting immune cell infiltration and function. The activation of the AIM2 inflammasome is involved in the establishment of an immunosuppressive lung microenvironment (106, 107), the recruitment of T cells and plasmacytoid dendritic cells (pDCs), as well as the polarization of tumor-associated macrophages (TAM) (**Figure 3**, right panels) (108, 109). AIM2 has also been observed to promote PD-L1 expression, suggesting that targeting AIM2 inflammasome therapy could potentially down-regulate PD-L1 expression, thereby counteracting the immune evasion in NSCLC (**Figure 3**, right panels) (101, 109, 110).

Research indicates that AIM2, predominantly localized within the mitochondria, exerts influences over mitochondrial morphology and reactive oxygen species (ROS) generation (103). AIM2 knockdown promoted mitochondrial derivation and fusion, whereas AIM2 upregulation promoted mitochondrial fission and fragmentation (103). Correspondingly, ROS produced by mitochondria fluctuate in response to changes in mitochondrial morphology, with, increased mitochondrial fission significantly heighten ROS levels in NSCLC cells (103, 111). Mitochondrial dynamics and functionality in mitochondria in cancer are pivotal to tumor progression, particularly through excessive production and alterations of ROS (**Figure 3**, right panels).

Intriguingly, AIM2 emerges as a distinctive biomarker of differential expression, uniquely identifying adenocarcinoma-like large cell lung cancer (112). There is an imperative for further research about the relationship between AIM2 and NSCLC (**Table 1**).

#### 3.2.3 AIM2 in breast cancer

Extensive research has shown the role of pyroptosis in the occurrence, progression, and prognosis of BC. AIM2 and Z-deoxyribonucleic acid-binding protein 1 (ZBP1) were found to be important pyroptosis genes in DEGs, and significant differences in their expression in primary lesions and BMs were observed. Patients with a high expression of AIM2 had a worse prognosis than low expression, while patients with a high expression of ZBP1 had a better prognosis than low expression. AIM2 and ZBP1 increase immune cell infiltration and may be potential targets for predicting and treating triple-negative breast cancer (TNBC) patients with brain metastasis (BM) (113).

#### 3.2.4 AIM2 in hepatocellular carcinoma

In the context of exploring the mechanisms underlying diethylnitrosamine (DEN)-induced HCC in murine models, recent research has highlighted the pivotal role of genetic inactivation of AIM2 in modulating liver pathology and tumorigenesis (114). Specifically, studies have demonstrated that the genetic inactivation of AIM2, but not NLRP3, results in a significant reduction in liver damage and the progression of HCC in this experimental paradigm. Notably, the deficiency of AIM2 appears to ameliorate the activation of the inflammasome, a critical component of innate immune responses, thereby mitigating liver inflammation and the proliferative responses that are integral to the initiation phase of HCC (114). These findings suggest a nuanced regulation of inflammatory pathways by AIM2, which may represent a promising therapeutic target for interrupting the carcinogenic process in DEN-induced liver cancer.

#### 3.2.5 AIM2 in renal cell carcinoma

The oncogenic role of AIM2 in RCC has also been reported, highlighting its complex and paradoxical functions. In scenarios of acute tubular necrosis, the AIM2 inflammasome is capable of detecting DNA amidst necrotic remnants, thereby triggering an onset of necrotizing inflammation (115). Additionally, studies have shown that AIM2 can promote RCC progression and resistance to therapies such as sunitinib, a common treatment for RCC, independently of the inflammasome. Specifically, AIM2 has been found to reduce the susceptibility of RCC to sunitinib through the FOXO3a-ACSL4 axis-regulated ferroptosis, a type of programmed cell death distinct from apoptosis (**Figure 3**, right panels and **Table 1**) (116). Collectively, the intricate and context-dependent role of AIM2 in RCC underscores its potential as both a driver of tumorigenesis and a therapeutic target, underscoring further investigation into the mechanisms by which AIM2 modulates RCC biology.

#### 3.2.6 AIM2 in gastric cancer

In a study leveraging the comprehensive resources of the TCGA database, coupled with primary GC samples from five patients, Feng et al. reported a significant upregulation of AIM2 expression within GC tissues (117). Their findings underscored the functional significance of AIM2, demonstrating that its knockdown effectively inhibited the invasive and migratory capabilities of GC cells by modulating and attenuating excessive MAPK signaling (**Figure 3**, right panels). This work contributes to a growing body of evidence indicating that AIM2 upregulation in GC tissues correlates adversely with patient survival outcomes. Further research has expanded our understanding of STAT3-mediated upregulation of the AIM2 interacting with microtubule-associated end-binding protein 1 (EB1) in GC, which facilitates epithelial cell migration and tumorigenesis, operating independently of inflammasome activition (**Figure 3**, right panels and **Table 1**) (118). These findings highlight AIM2 as a pivotal player in GC progression, linking innate immune responses and cellular migration to oncogenic processes. The identification of AIM2 as a key mediator in these pathways not only enhances our understanding of GC biology but also presents potential therapeutic targets for interventions aimed at mitigating GC progression and improving patient outcomes.

## 4 The dual role of IFI16 in cancer

### 4.1 The anti-tumor role of IFI16

#### 4.1.1 IFI16 in squamous cell carcinoma

**Figure 4:**
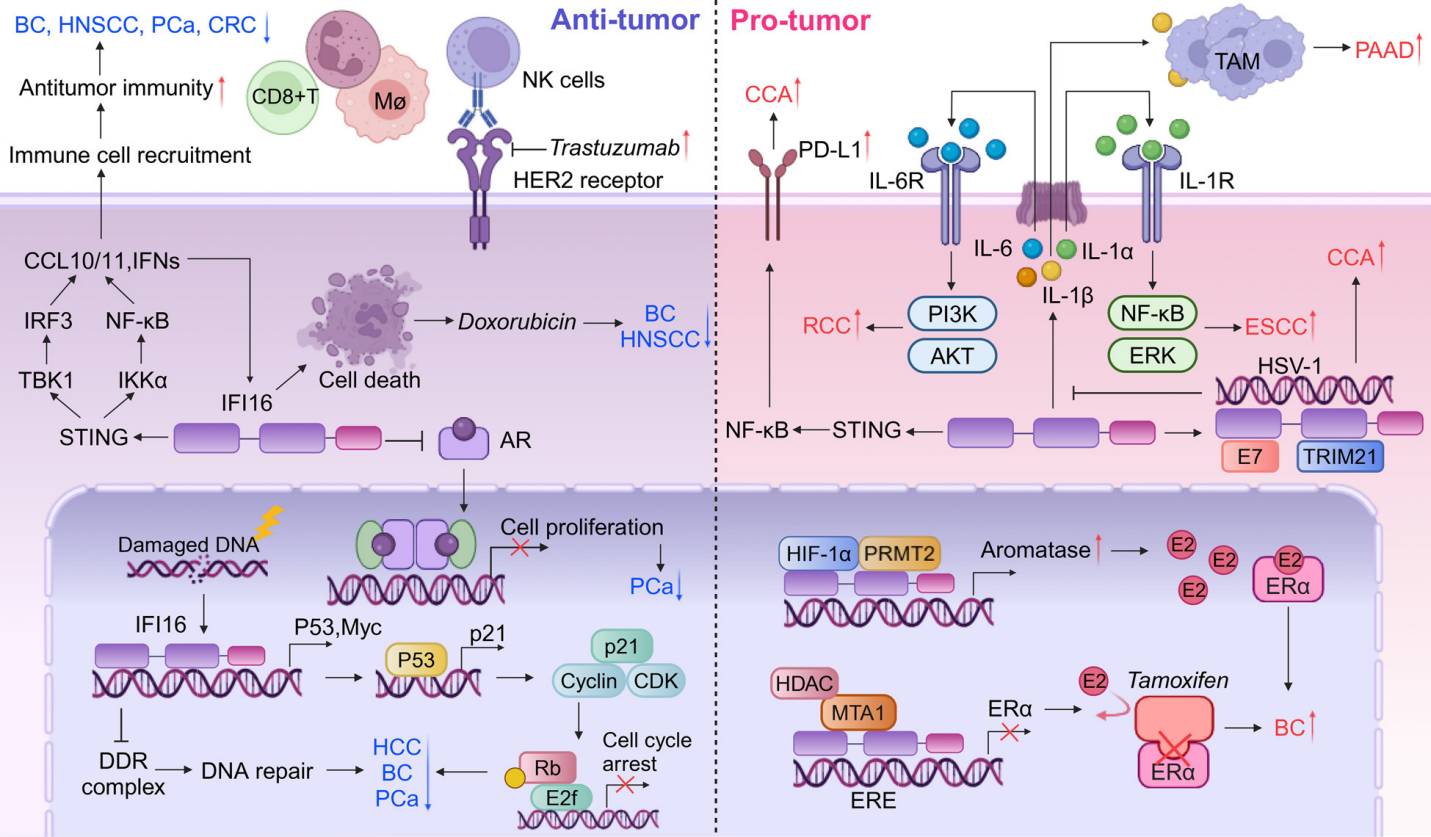
Dual roles of IFI16 in tumor microenvironment. IFI16 functions as a critical mediator in the tumor microenvironment, linking DNA damage to subsequent antitumor responses. It inhibits DNA repair processes, thereby promoting cell cycle arrest associated with cellular senescence and increasing cellular vulnerability to further DNA damage. The activation of the IFI16-STING pathway plays a central role in orchestrating antitumor immune responses, exhibiting tumor-suppressive properties and enhancing the chemosensitivity of cancer cells to therapeutic agents such as trastuzumab and doxorubicin. Conversely, IFI16 can also contribute to inflammasome formation, leading to the release of various inflammatory cytokines. These cytokines can activate intracellular signaling pathways in an autocrine manner or recruit TAMs, which may promote oncogenic progression and create an immunosuppressive microenvironment. Furthermore, IFI16 is involved in modulating hormone receptors, including AR and ER, thereby exerting diverse effects on tumor biology that range from suppression to promotion.

Restoration of IFI16 levels in head and neck squamous cell carcinoma (HNSCC) exhibits potent antitumor effects by inhibiting tumor growth and *in vitro* transforming activity, as well as augmenting doxorubicin-induced cell death via the accumulation of cells at the G2/M phase (**Figure 4**, left panels and **Table 2**) (119). Subsequent investigations have further elucidated the mechanisms underlying IFI16’s antitumor activity *in vivo*. Specifically, IFI16 promotes apoptosis of tumor cells, inhibits neovascularization, and enhances the recruitment of CD68/CD14-positive macrophages through the release of chemotactic factors (**Figure 4**, left panels and **Table 2**) (120). Intriguingly, a correlation has been observed between IFI16 expression and the prognosis of HPV-positive HNSCC cases with low proliferative indices. Specifically, tumors positive for IFI16 staining and exhibiting high levels of the tumor suppressor protein pRb were associated with a more favorable prognosis compared to those with low IFI16 and pRb levels and a high proliferation index (121). This finding underscores the potential of IFI16 as a biomarker for predicting disease outcome in this subset of HNSCC patients. Moreover, an additional layer of complexity in the role of IFI16 in HNSCC has been revealed by studies indicating a negative correlation between IFI16 expression and lymph node metastasis, even in the absence of HPV infection (122). This observation suggests that IFI16 may play a protective role against lymphatic dissemination of HNSCC cells, irrespective of the HPV status of the tumor. Future research is needed to further elucidate the molecular mechanisms underlying IFI16’s antitumor activities and to explore its clinical application in the management of HNSCC patients.

**Table 2:** The role of IFI16 in tumor biology and the underlying mechanisms in various tumor types.

Cancer types	Molecular mechanisms of IFI16	System	Role of IFI16	Reference
SCC	Increasing doxorubicin-induced cell death by enhancing G2/M phase arrest	HNSCC derived cell line HNO136,	Antitumor	(119, 120)
	Inducing IL-1α to promote tumor progression through the Erk and NF-κB signaling	Human ESCC cell lines TE-9, TE-10, and TE-11	Protumor	(126)
	Upregulating FGF protein to promote metastasis	Human ESCC cell lines (KYSE30, KYSE180, KYSE450) and high metastasis model 30M cells	Protumor	(138)
BC	Enhancing trastuzumab response in HER2-positive breast cancer by activating the STING cascade	HER2 + cell line (SKBR3, MDA-MB-361, BT-474), TNBC cell line (MDA-MB-231, MDA-MB-436), breast epithelial cell line (MCF10A)	Antitumor	(126)
	Mediating BC cell proliferation and migration via HERC 5 / IFI 16 / p53 signaling	MCF-7 and MDA-MB-231 BC cells	Antitumor	(125)
	Binding to toll-like receptor 2 and inducing macrophages to secrete proinflammatory cytokines	BC cell lines (MDA-MB-231, BT549, Hs578T, BT-474, MCF7, T47D,4T1, E0771, 67NR), monocytic cell line (THP-1), and kidney epithelial-like cell line (HEK293T)	Protumor	(124)
	Promoting aromatase expression and E2 production in the surrounding adipose tissue	MCF7, T47D, BT474 and THP1 cell line	Protumor	(139)
HCC	Inducing tumor inhibition via activation of p53 signals and inflammasome	SMMC-7721 and Huh7 HCC cells	Antitumor	(127, 143)
	Promoting DNA damage through interaction with IRF3 and reversing cisplatin resistance	Huh7 and HepG3 HCC cells	Antitumor	(9)
	Regulated by DLK1 to induce cell proliferation *in vitro*	SMMC-7721 and Huh7 HCC cells	Protumor	(141)
RCC	Promoting RCC progression through the IL6/PI3K/Akt axis	Human RCC cell lines (769-P, 786-O, ACHN, Caki-1, OS-RC-2) and human proximal tubular epithelial cell line (HK-2)	Protumor	(145)
CRC	Positively correlated with the expression of Ki67	CRC tissues and adjacent normal tissues	Antitumor	(129)
	Associated with the RAS pathway	HCT116 and RKO CRC cells	None	(131, 132)
PCa	Down-regulating AR expression and inhibiting cell proliferation	LNCaP PC cells	Antitumor	(134)
HPV‐infected CCA	Regulating the expression of PD-L1 by promoting the activation of the STING-TBK1-NF-κB pathway	Human HPV-positive cervical cancer cell lines (CaSki, SiHa, Hela) and HPV-negative cervical cancer cells (C33A)	Protumor	(148)

#### 4.1.2 IFI16 in breast cancer

The tumor-suppressive functions of IFI16 are closely linked to its unique DNA-binding capacity, particularly its interactions with single-stranded DNA (ssDNA) and cruciform structures. Notably, clinical observations reveal reduced IFI16 expression levels in BC tissues, reinforcing its crucial role as a tumor suppressor intriguing DNA-binding capability of the IFI16 protein towards ssDNA and cruciform structures emerges as a pivotal aspect in its tumor suppressive functions (123). Interestingly, while IFI16 primarily exerts its tumor-suppressive effects through nuclear DNA surveillance, elevated serum levels of this protein have paradoxically been associated with poor prognosis in BC patients (124). This clinical paradox suggests that extracellular IFI16 may execute non-canonical biological functions distinct from its established nuclear tumor-suppressive mechanisms. The interaction between IFI16 and p53 is more intricate than previously understood. IFI16 is traditionally recognized for its role in disrupting apoptotic pathways by modulating both the p53/p21^CIP1^ and Rb/E2F signaling axes. Notably, it establishes a self-amplifying regulatory loop through p53-mediated induction of its own expression, collectively driving malignant transformation. This constitutes a component of a positive feedback loop between p53 and IFI116 (**Figure 4**, left panels). However, study indicates that IFI16 may suppress p53 transcriptional activity in MCF-7 and MDA-MB-231 cell lines, while paradoxically increasing the expression of p53 mRNA at the post-transcriptional level. This complexity suggests a nuanced role for IFI16 in modulating p53 function, with potential implications for cancer development and progression. IFI16’s proteasomal degradation mediated by oncogenic factor HERC5 activates p53 transcription and enhances BC cell proliferation and migration (125). More recent insights have unveiled a novel facet of IFI16/Ifi202 within TME, where it triggers tumor-promoting inflammation, thereby contributing to the formation of an immunosuppressive TME in BC (124). This finding expands our understanding of IFI16’s multifaceted functions beyond its direct tumor suppressive activity. Triple-negative breast cancer (TNBC), characterized by the absence of progesterone receptor, ER, and HER2 expression, is a heterogeneous and basal-like subtype. In TNBC patients, high expression levels of IFI16 have been notably associated with improved outcomes following chemotherapy treatments. Specifically, these high levels potentiate the inhibitory effects of doxorubicin on tumor growth *in vivo (* 8). IFI16 amplifies the STING-induced type I IFN response by suppressing DNA repair and facilitating the translocation of DNA fragments into the cytoplasm, thereby intensifying the antitumor activity in TNBC (**Figure 4**, left panels). This suggests that IFI16 may serve as a sentinel and mediator of DNA damage, orchestrating subsequent antitumor responses within the TNBC microenvironment (8). Furthermore, for human epidermal growth factor receptor 2 (HER2)-positive BC, IFI16 is a key determinant in the response to monoclonal antibody therapy by activating the STING cascade (126). The reactivation of IFI16-directed immune responses has the potential to convert HER2-positive breast cancer into immunologically active ‘hot tumors’, thereby augmenting patient responsiveness to trastuzumab treatment (**Figure 4**, left panels and **Table 2**) (126).

Collectively, these findings underscore the nature of IFI16 in BC, with its tumor suppressive properties being counterbalanced by its potential to elicit an immunosuppressive TME.

#### 4.1.3 IFI16 in hepatocellular carcinoma

IFI16 expression is significantly downregulated in HCC tissues, highlighting its potential role as a tumor suppressor. Restoration of IFI16 expression in HCC cells not only elicits p53/p21-dependent inhibition of tumor growth but also promotes the recruitment of inflammasomes, suggesting its multifaceted antitumor effects (**Figure 4**, left panels) (127). Furthermore, studies have demonstrated that IFI16 enhances chemosensitivity to cisplatin and increases susceptibility to DNA damage, thereby augmenting the efficacy of chemotherapy in HCC (9). Collectively, these findings underscore the importance of IFI16 as a tumor suppressor in HCC and its potential therapeutic implications (**Table 2**).

#### 4.1.4 IFI16 in colorectal cancer

The phenotypic expression characterized by IFI16 negativity combined with Ki-67 positivity (IFI16-/Ki-67+) has been notably and positively correlated with advanced TNM staging in cancer patients, and it exhibits a borderline significant link to lymph node metastasis, as reported in a pivotal study (**Table 2**) (128, 129). Intriguingly, the expression levels of IFI16 did not display any statistically significant association with the infiltration of CD8+ TILs or the expression of PD-L1, implying that its impact on cancer progression may be independent of these immunological factors. Factors causing DNA damage, such as oxidative stress, promote the deubiquitination of IFI16, thereby driving the STING-mediated antitumor immune response in CRC (**Figure 4**, left panels) (130). It is noteworthy that IFI16 also acts as an RAS pathway related protein, which frequently activates mutations in CRC (131, 132). Given the close association between RAS pathway activation and tumor proliferation as well as invasiveness, it warrants further investigation to elucidate its potential as a therapeutic target. Collectively, the findings from this research endeavor offer a fresh perspective on the functional role of IFI16 in the pathogenesis of CRC, particularly through its modulation of cancer cell proliferation. This novel insight underscores the potential significance of IFI16 as a biomarker and therapeutic target in CRC management.

#### 4.1.5 IFI16 in prostate cancer

IFI16 has emerged as a pivotal regulator of cellular senescence and growth arrest in PCa cell lines. By upregulating p21WAF1 and inhibiting E2F-stimulated transcription, IFI16 suppresses cellular proliferation (133). Elevated levels of IFI16 in prostate epithelial cells (PrECs) contribute to senescence-associated growth arrest, suggesting its role in PCa suppression. IFI16 binds to the androgen receptor (AR) in a ligand-dependent manner, specifically through the AR’s DNA-binding domain (DBD) (**Figure 4**, left panels and **Table 2**) (134). Re-expression of IFI16 in LNCaP cells, which lack endogenous IFI16, downregulates AR expression and inhibits the expression of AR target genes, leading to decreased cell viability and apoptosis (135). These findings support the hypothesis that histone deacetylase-dependent transcriptional silencing of IFI16 contributes to PCa development. Importantly, the re-expression of IFI16 orthologs in immunocompetent mice abrogates tumorigenesis and activates anti-tumor immunity, indicating that early epigenetic changes may influence tumorigenesis by targeting co-located genes (**Figure 4**, left panels) (136). Hypomethylation domains are detectable in blood samples enriched for metastatic circulating tumor cells, providing insights into the potential mechanisms underlying PCa progression.

#### 4.1.6 IFI16 in osteosarcoma

In human OS, the expression level of IFI16 is markedly reduced compared to that in normal bone tissue. Overexpression of IFI16 has been demonstrated to modulate the expression of several key genes involved in cell cycle regulation and oncogenesis. Specifically, it upregulates p21 while downregulating cyclin E, cyclin D1, as well as the oncogenes c-Myc and Ras (137). In the context of viral infections, loss of IFI16 has been shown to impact chromatin labeling in OS cells, characterized by increased labeling of active chromatin and decreased labeling of viral and cellular gene promoters that inhibit chromatin activity (40). These findings suggest that IFI16 plays a pivotal role in histone modification and the regulation of gene expression in OS cells, potentially influencing the epigenetic landscape and DNA accessibility for transcriptional regulation.

### 4.2 The pro-tumor role of IFI16

#### 4.2.1 IFI16 in squamous cell carcinoma

In ESCC, the role of IFI16 appears to contrast with its function in HNSCC. Specifically, high expression levels of IFI16 within ESCC tissues have been associated with unfavorable prognostic outcomes and increased macrophage infiltration, indicating its potential as a marker of aggressive disease (126). Mechanistically, IFI16-induced IL-1α has been shown to exacerbate the malignant phenotype of ESCC cells, with particular impacts on cell survival and migration, thus contributing to disease progression (**Figure 4**, right panels) (126). Furthermore, emerging evidence suggests that IFI16 promotes ESCC metastasis by upregulating the expression of fibroblast growth factor (FGF) proteins, which are known to play crucial roles in cancer cell migration, invasion, and angiogenesis (138). These findings (**Table 2**) underscore the complex and context-dependent roles of IFI16 in different cancer types and highlight its potential as a therapeutic target in ESCC.

#### 4.2.2 IFI16 in breast cancer

In the context of estrogen receptor (ER)-positive BC, IFI16 plays a pivotal role in augmenting tumor growth by facilitating the transcriptional activation of aromatase and subsequent E2 production in adipose tissue (**Figure 4**, right panels and **Table 2**) (139). Intriguingly, a stable knockdown of IFI16 or MTA1 in an MDA-MB-231-derived line significantly sensitized these cells to tamoxifen-induced growth inhibition. These findings collectively suggest that the MTA1-TFAP2C or MTA1-IFI16 complex may be involved in the epigenetic regulation of ESR1 expression, thereby influencing the chemosensitivity of BC tumors to tamoxifen therapy (140).

#### 4.2.3 IFI16 in hepatocellular carcinoma

Delta-like 1 (DLK1) upregulated the expression of IFI16 and its promoter transcriptional activity, is accompanied by a decrease in p21^WAF1/CIP1^ levels and subsequent acceleration of the cell cycle, suggesting that IFI16 may serve as a pivotal downstream target of DLK1 in promoting HCC cell proliferation (**Table 2**) (141). Furthermore, the chromatin-bound localization of IFI16 has been implicated in the progression of HCC, indicating its role as a potential biomarker or therapeutic target in this disease (142). Of note, Nutlin-3, a DNA damage agent, has been found to alter the subcellular localization of chromatin-bound IFI16 in HCC cells *in vitro*. This re-localization appears to be regulated in a p53-dependent manner, suggesting that Nutlin-3 may exert its effects on HCC cells through modulation of the p53-IFI16 axis (143). These findings provide new insights into the complex regulatory networks governing HCC progression and highlight the potential of targeting IFI16 and its interactions with other proteins, such as p53 and DLK1, for therapeutic intervention in this disease.

#### 4.2.4 IFI16 in renal cell carcinoma

Elevated levels of IFI16 have been observed to positively correlate with several key clinicopathological features of RCC, including lymphatic metastasis, tumor stage, and histopathological grade. Notably, high IFI16 expression has been linked to adverse patient prognosis, with studies indicating that such expression is predictive of worse overall survival outcomes (144). At the molecular level, IFI16 has been shown to play a pivotal role in promoting clear cell RCC (ccRCC) progression. Specifically, IFI16 stimulates the transcription and translation of IL-6, which in turn activates the PI3K/Akt signaling pathway and induces EMT (**Figure 4**, right panels). Indeed, studies have demonstrated that IFI16-induced EMT promotes the progression of clear cell RCC (ccRCC) (**Table 2**) (145). Together, these findings provide new insights into the mechanisms underlying RCC progression and highlight the potential of targeting IFI16-related pathways for therapeutic intervention in this disease.

#### 4.2.5 IFI16 in pancreatic adenocarcinoma

Overexpression of IFI16 has been implicated in the activation of the inflammasome, leading to the induction of IL-1β production in the tumor microenvironment of PAAD. This IL-1β production, in turn, mediates the maturation, proliferation, and migration of tumor-associated macrophages (TAMs), ultimately promoting the growth and progression of PAAD tumors. Notably, downregulation of IFI16 has been shown to reduce the infiltration of TAMs in the PAAD microenvironment when treated with gemcitabine, and to enhance the sensitivity of gemcitabine in these tumors (**Figure 4**, right panels) (146). Taken together, these findings suggest that IFI16 may emerge as a pivotal target for the development of novel therapeutic strategies aimed at inhibiting PAAD progression and improving treatment efficacy.

#### 4.2.6 IFI16 in HPV-infected cervical cancer

The expression of IFI16 is significantly higher in HPV-positive lesions compared to HPV-negative lesions (147), and positively correlated with HPV infection (148). IFI16 has been implicated in positively regulating the expression of PD-L1 via the STING-TBK1-NF-κB signaling pathway, thereby fostering the progression of CCA, as evidenced in a recent study (**Figure 4**, right panels and **Table 2**) (148). This revelation underscores the intricate roles that IFI16 plays in the oncogenic processes of CCA and suggests its potential as a novel therapeutic target for immunotherapy in the future. Furthermore, intriguing interactions between the HPV E7 oncogene and both IFI16 and TRIM21 have been documented (149). Specifically, E7 interacts with these proteins, leading to the recruitment of the E3 ligase TRIM21, which in turn ubiquitinates and degrades the IFI16 inflammasome (**Figure 4**, right panels). This process results in the inhibition of cell pyroptosis and enables cancer cells to evade immune surveillance (149). These findings warrant further investigation into the multifaceted roles of IFI16 in CCA progression and highlight its potential as a key player in the modulation of immune responses in this malignancy.

## 5 The anti-tumor role of IFIX

### 5.1 IFIX in breast cancer

In the context of breast tumorigenesis, the expression of IFIX is frequently downregulated, serving as a pivotal tumor suppressor. Despite its recognized role, the intricate mechanisms underlying IFIX’s antitumor effects remain an area of ongoing research. Several plausible pathways through which IFIX exerts its suppressive influence on BC progression can be delineated as follows: Firstly, IFIXα1 exhibits an autonomous tumor-suppressive function that transcends the classical regulatory pathways involving the pRb and p53. Specifically, IFIXα1 upregulates the expression of p21CIP1, a cyclin-dependent kinase (CDK) inhibitor. This upregulation leads to a reduction in the kinase activities of Cdk2 and p34Cdc2, the latter being a crucial CDK controlling the G2/M transition phase of the cell cycle. Consequently, the activation of p34Cdc2, which is essential for the progression through S-phase and the G2/M transition, is impeded, resulting in the accumulation of cells in these phases (150). Secondly, IFIX negatively regulates HDM2 (also known as MDM2), a protein implicated in oncogenesis. HDM2 functions by inhibiting the activity of p53 through binding to its N-terminus. Following DNA damage, an intriguing shift occurs in the ubiquitination process mediated by MDM2’s E3 ubiquitin ligase activity. Specifically, ubiquitination is partly redirected from p53 to MDMX, thereby facilitating p53 activation. This intricate p53/MDM2 feedback loop plays a critical role in maintaining appropriate p53 levels within the cell (19). Thirdly, IFIXα is also implicated in the downregulation of histone deacetylase 1 (HDAC1) protein via a ubiquitin–proteasome pathway. This downregulation has indirect consequences on BC cell behavior, specifically promoting the transactivation of maspin in MDA-MB-468 cells (151). The modulation of HDAC1 by IFIXα represents an additional layer of complexity in its anti-tumor mechanisms, highlighting its multifaceted role in BC suppression. Collectively, these findings underscore the significant anti-tumor potential of IFIX in BC and suggest multiple mechanisms through which it exerts its suppressive effects.

### 5.2 IFIX in oral squamous cell carcinoma

The recently discovered IFIX has emerged as a pivotal tumor suppressor in breast cancer. However, our understanding of IFIX’s role in OSCC remains relatively nascent. Intriguingly, studies have demonstrated that the overexpression of IFIX potently inhibits the invasive capabilities of human CAL-27 cells. This inhibitory effect is mechanistically underpinned by the stabilization of the cytoskeleton, achieved through the modulation of various cytokeratin (152). Additionally, IFIX downregulates paxillin, an intracellular adaptor protein known to facilitate tumor invasion (152). Notably, the stabilization of the cytoskeleton is closely linked to the suppression of EMT, which involves extensive cytoskeletal reorganization to enable cancer cells to acquire migratory and invasive properties. In this context, IFIX has been shown to inhibit EMT by mediating the Wnt/β-catenin signaling pathway through the upregulation of naked cuticle 2 (NKD2), a known inhibitor of this pathway in CAL-27 cells (153). By suppressing Wnt/β-catenin signaling, IFI16 prevents the transcriptional activation of EMT-inducing genes, thereby maintaining epithelial characteristics and reducing tumor aggressiveness (153). Collectively, these findings contribute novel and fundamental insights into the tumor-suppressive functions of IFIX, particularly its role in stabilizing the cytoskeleton of cancer cells and preventing the transition to a mesenchymal phenotype.

## 6 The dual role of MNDA in cancer

### 6.1 The anti-tumor role of MNDA in myelodysplastic syndromes

The human MNDA, a nuclear protein specific to myelomonocytic cells, is regulated by interferon alpha in a cell-specific manner. MNDA influences the function of ubiquitous proteins like nucleolin in a cell/lineage- and differentiation-specific context (154). Elucidating MNDA’s protein binding could advance our understanding of its function and the roles of other interferon-inducible gene family members. MNDA interacts with multiple proteins regulating gene transcription, exhibiting unique expression patterns across cell lineages and mediating interferon effects (155). Notably, MNDA alters the expression of a hematopoiesis-essential gene (155). Reduced MNDA transcripts are observed in both familial and sporadic MDS cases (156, 157), impacting programmed cell death in myeloid progenitor cells and enhancing tumor necrosis factor-related apoptosis inducing ligand (TRAIL)-induced cell death sensitivity in granulocyte-macrophage progenitors (158). Lower MNDA expression in granulocytes and blasts distinguishes MDS from non-MDS, establishing it as an independent marker for dyspoiesis assessment and a potential addition to flow cytometric (FCM)-based quantitative panels (159). In summary, MNDA’s multifaceted role in regulating cellular processes, particularly within the myelomonocytic lineage, and its potential implications in diseases such as MDS, underscore the necessity for further investigation into its protein interactions and functional mechanisms. Such endeavors could significantly contribute to advancing our understanding of hematopoiesis and the pathobiology of MDS.

### 6.2 The pro-tumor role of MNDA in marginal zone lymphoma

In recent scientific inquiries, the expression profile of MNDA has emerged as a significant biomarker in the context of various B-cell lymphomas. MNDA is constitutively expressed in a distinct subset of marginal zone B cells within normal tissue, underscoring its physiological role in B-cell biology. Notably, this expression pattern extends to specific lymphoma subtypes, particularly those originating from the marginal zone, such as mucosa-associated lymphoid tissue (MALT) lymphoma, splenic marginal-zone lymphoma (SMZL), and nodal marginal zone lymphoma (NMZL). The preferential expression of MNDA in these lymphomas, coupled with its rarity in follicular lymphoma (FL), positions it as a valuable diagnostic tool in differentiating NMZL from FL (160). Moreover, MNDA’s elevated expression in MZL further enhances its utility in distinguishing MZL from reactive lymphoid hyperplasia (RLH) and FL. However, its discriminatory power wanes when distinguishing MZL from mantle-cell lymphoma (MCL) and chronic lymphocytic leukemia/small lymphocytic lymphoma (CLL/SLL) (161). Intriguingly, within diffuse large B-cell lymphoma (DLBCL), MNDA expression is more prevalent in the non-germinal center B-cell (non-GCB) subtype and the BCL2/MYC double-expression group, displaying a correlation with CD5 expression—a finding that merits further exploration. The clinical implications of MNDA expression and its potential prognostic significance in these lymphomas remain incompletely understood and warrant thorough elucidation. Interestingly, MNDA expression does not serve as an effective identifier in cases characterized by MYD88 p.Leu265Pro mutations or specific SMZL-related genetic aberrations (162), indicating that its diagnostic utility is context-dependent. A novel insight comes from the VR09 cell line, derived from an atypical, non-CLL B-cell chronic lymphoproliferative disorder with plasmacytic features. This cell line exhibits an activated B-cell maturation phenotype with secretory differentiation, harboring an episomal EBV genome, trisomy of chromosome 12, and a wild-type p53 status, among other genetic features. Notably, it also displays MNDA expression, suggesting that this EBV-positive cell line could serve as a valuable model for further characterizing *in vivo* activated DLBCL with plasmacytic characteristics (163).

In summary, MNDA expression profiling has emerged as a promising diagnostic aid in the differentiation of specific B-cell lymphoma subtypes, particularly within the context of MZL and certain DLBCL subsets. However, its application should be nuanced, considering its variable expression across different lymphoma entities and its limitations in certain genetic contexts. Future studies are needed to fully elucidate the clinical relevance and prognostic implications of MNDA expression in these lymphomas.

### 6.3 The anti-tumor role of MNDA in osteosarcoma

MNDA was highly expressed in normal bone tissue, but was significantly lower in OS cells (164). MNDA overexpression effectively inhibited proliferation induced apoptosis and reduced migration/invasiveness in Saos-2 cells (164). To further explore the potential mechanisms underlying MNDA’s tumor-suppressive effects, recent studies have identified hsa-miR-889-3p as a key regulator. This microRNA could promote the proliferation of OS through inhibiting MNDA expression, which provides a potential therapeutic strategy in treatment for OS (165). In the tumor microenvironment of OS, MNDA+ macrophages could also contribute to disease progression by enhancing osteoclast activity, leading to bone destruction and the release of growth factors that promote tumor cell proliferation and invasion (166). Therefore, targeting MNDA+ macrophages, restoring MNDA expression in OS cells, or inhibiting hsa-miR-889-3p could represent promising therapeutic strategies for OS, particularly in preventing bone destruction and tumor progression.

### 6.4 The pro-tumor role of MNDA in hepatocellular carcinoma

MNDA, predominantly expressed in tumor-associated M2 macrophages, critically orchestrates the crosstalk between M2 macrophages and HCC cells through exosome-mediated transfer of pro-metastatic proteins (167). Mechanistically, MNDA enhances M2 macrophage polarization by upregulating key polarization enhancers such as TGFB1, CSF1, and S100A4. Furthermore, MNDA drives the selective packaging of metastasis-promoting proteins, including NRP2, TIMP1, ITGB2, and ITGAM, into exosomes secreted by M2 macrophages. M2-derived exosomes robustly enhance HCC cell migration, an effect attenuated by MNDA knockdown (167). Clinically, high MNDA expression correlates with poor prognosis in HCC patients, highlighting its potential as a therapeutic target. Targeting MNDA or its exosomal effectors could disrupt M2-TAM protumor functions and ameliorate the immunosuppressive TME, offering a novel strategy to impede HCC progression.

## 7 Conclusion and perspective

**Figure 5:**
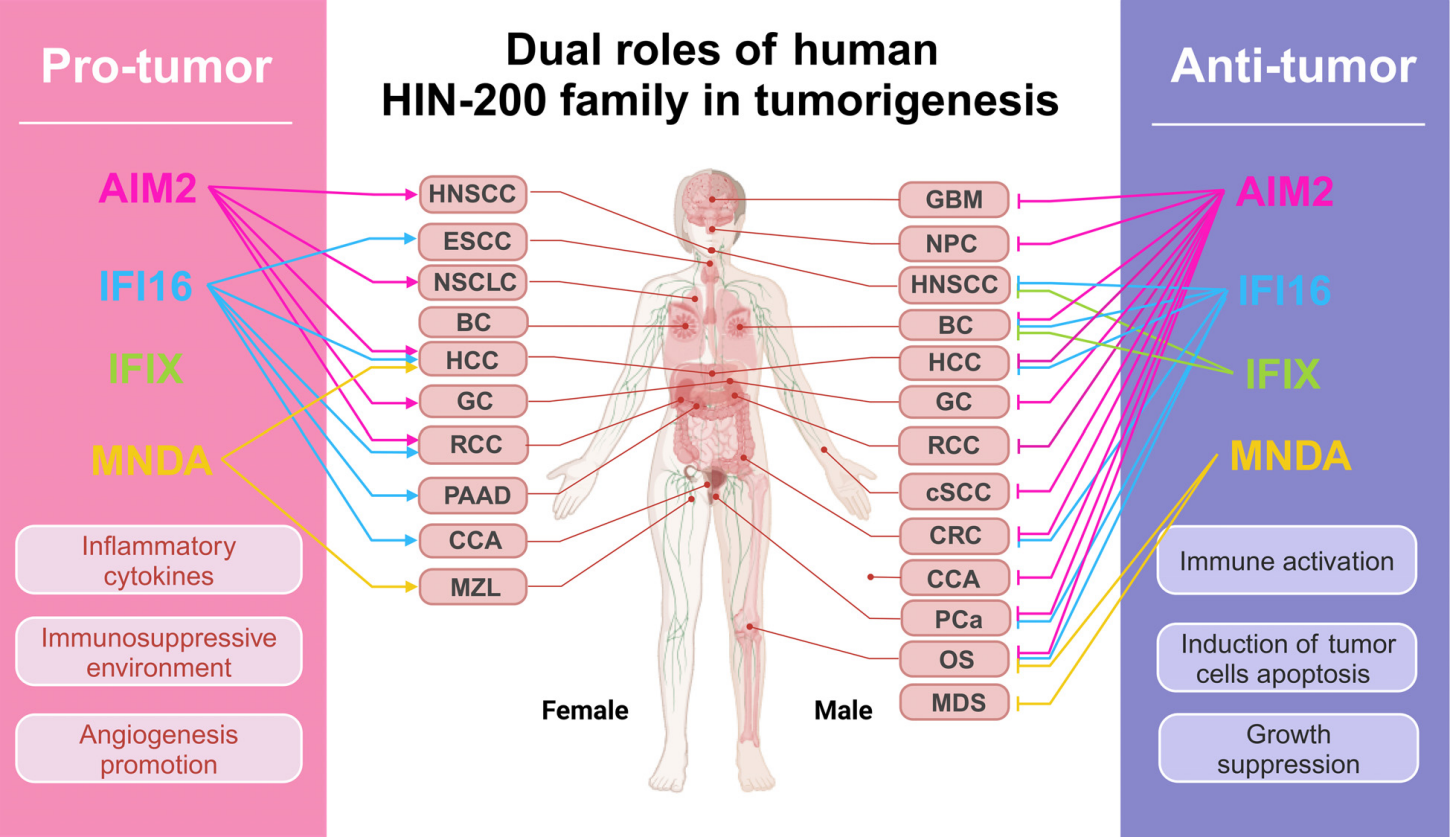
Dual roles of human PYHIN family in tumorigenesis. The figure encapsulates the impact of PYHIN family molecules on common human cancers, as detailed in the article. In the context of pro-active cancers, these molecules facilitate cancer cell metastasis and immune evasion by fostering an inflammatory and immunosuppressive milieu. Conversely, they impede tumor advancement by activating intrinsic immunity and triggering apoptosis in cancer cells. All oncological terminology abbreviations are spelled out upon their first mention in the text.

The PYHIN family proteins have emerged as pivotal regulators in the intricate interplay between immune responses and cellular homeostasis, with their dysfunction intricately linked to the pathogenesis of numerous malignancies. The dualistic nature of inflammasomes in cancer (**Figure 5**), driven by tumor heterogeneity and the TME plasticity, underscores the complexity of their roles. While they can foster a pro-tumorigenic environment by promoting angiogenesis and recruiting immunosuppressive cells (168), they can also elicit an anti-tumorigenic response by activating immune cells and enhancing the elimination of tumor cells through pyroptosis (101, 109). Intriguingly, AIM2 and IFI16, despite their ability to activate inflammasomes and influence the TME, may play differing or even opposing roles within the same tumor. In inflammation-driven cancers like CRC, they typically function as tumor suppressors (74, 75, 129). Conversely, in HCC, a different inflammation-related cancer, AIM2 adopts an oncogenic role while IFI16 retains its tumor-suppressive properties. Despite their distinct functions, they are interdependent within the microenvironment, influencing each other’s activities. Notably, IFI16 plays a crucial role in modulating inflammation by inhibiting caspase-1 activation in both AIM2 and NLRP3 inflammasomes (133). Furthermore, in the context of PANoptosis, an inflammatory cell death pathway involving AIM2 and other inflammasomes, IFI16 emerges as a key regulatory factor (169). The complex interplay between AIM2 and IFI16 inflammasomes and its implications for disease pathogenesis warrant further investigation, as the relationship between the PYHIN family and cancer holds significant, yet untapped, potential.

Beyond their canonical roles in inflammasome formation, PYHIN family proteins exhibit diverse inflammasome-independent functions in cancer. For instance, AIM2 regulates intestinal stem cell proliferation and gut microbiota, influencing CRC pathogenesis (74, 75). IFI16, by sequestering transcription factors and modulating the expression of viral and cellular genes, impacts cancer progression (170). Furthermore, IFIX’s ability to detect viral DNA and induce an interferon response hints at its potential protective role against virus-induced cancers (126, 133). MNDA, typically linked to hematological disorders characterized by apoptotic imbalances, has been implicated in promoting OS metastasis (167), suggesting that the PYHIN family may have novel roles in a broader range of tumors. The development of broader and more effective anticancer drugs is a promising avenue.

Current strategies focus on molecularly tailored interventions against PYHIN family members through multiple modalities. Small-molecule inhibitors, like quercetin and Rg1, suppress inflammasome activity, mitigating treatment-related toxicity from radiotherapy or chemotherapy (171, 172), while PYHIN agonists, such as deoxyribonuclease 1 like 3 (DNASE1L3) and dihydroartemisinin, may enhance therapeutic efficacy in specific contexts (53, 60, 173). Tissue-targeted viral and non-viral gene delivery systems enable precise modulation of PYHIN expression to inhibit tumor progression (65, 174). Combination approaches integrating DNA sensor targeting with immune therapy or radiation therapy demonstrate synergistic antitumor effects, creating a promising therapeutic blueprint (58, 148, 175, 176). However, challenges remain, such as systemic toxicity of inflammasome suppression, which may compromise innate immunity or exacerbate off-target effects. Optimized combinatorial regimens balancing inflammasome modulation with conventional therapies, coupled with dynamically responsive drug delivery platforms, are essential to maximize therapeutic windows and minimize adverse outcomes.

Despite the significant progress made in understanding the roles of PYHIN family proteins in cancer, several key questions remain unanswered. For instance, future research should focus on elucidating the precise mechanisms underlying the dual roles of AIM2 and IFI16 in various cancers, as well as the interactions and crosstalk among PYHIN family members. Additionally, the potential synergistic effects of targeting multiple PYHIN family members or combining PYHIN-targeted therapies with other treatment modalities (such as immunotherapy, chemotherapy, or radiation therapy) need to be explored. Clinical trials are essential to validate the efficacy and safety of these therapies in patients with different cancer types. In short, although further work is needed, the PYHIN family proteins represent promising targets for cancer therapy, and continued research in this area holds great potential for improving patient outcomes.
